# Notes and News

**Published:** 1886-12-04

**Authors:** 


### Notes and News.

Collected and Communicated.

H.R.H. Princess Christian has consented to become patroness of the After Care Association for Poor Female Mental Convalescents, of which Lord Brabazon is the President. The Committee are anxious to open a Convalescent Home at once, for which purpose about £1,000 is required, and help from the charitably-inclined is therefore earnestly requested. All information may be obtained from the Secretary, Mr. H. Thornhill Roxby, Emblewood, Osbaldeston Road, N.

The Duke of Northumberland has promised a site for the proposed new infirmary buildings at North Shields, and Mr. R. S. Donkin, M.P., has given 200 guineas towards the cost of erection.

It has been decided to insert a stained glass window in the Royal College of Veterinary Surgeons, Didcot, in commemoration of the Queen's Jubilee.

A sum of £75 has been handed to the treasurer of the Princess Frederica's Convalescent Hospital, the amount of the offertories at St. Jude's, South Kensington, on Sunday week.

"The Mayor's Collection," in aid of the Stockport Infirmary, amounts this year to £120, the largest sum, with two exceptions, that has been raised by this means since 1874, when £206 17*s*. was collected.

Mr. Daniel Wood of Glossop has offered to build a hospital at a cost of £5,000, and to endow it with £20,000, and Mr. and Mrs. Samuel Wood will give and endow public baths to the town. Moreover, Captain Partington and Mr. Herbert Rhodes have each given £2,000 towards the institution of a free library and a town hall. Glossop at any rate has no cause to complain of want of generosity on the part of its more wealthy inhabitants.

Messrs. Sargant and Langford, of St. Bartholomew's Hospital College have been awarded the Preliminary Scientific Exhibition of £50 for one year, open to students of less than six months' standing. They were bracketed equal.

Mr. W. F. Browell has been unanimously elected president of the Tunbridge Wells Provident Dispensary, an office rendered vacant a short time since by the death of the Hon. F. G. Molyneux.

The Governors of the St. Alban's Hospital have decided to decline Mr. Blundell Maple's offer of a site for their new buildings, on the ground of its too close proximity to the Schools, and the Church; the noise of the one, and the bells (twelve in number) of the other, being in their opinion likely to irritate patients, and retard their recovery. We certainly agree with the Governors on these points, although it is unfortunate that so generous an offer should have to be rejected.

THE authorities of the Brompton Hospital have decided to keep a certain number of trained nurses to take charge of private patients, as numerous applications have proved that the want of such assistance is widely felt. By this arrangement, fully qualified nurses will be always available to undertake private cases of all sorts, whether medical or surgical, and nurses qualified in the practice of massage will also be obtainable on application to the Secretary.

---

THE following vacancies are announced this week, the amount of the salary being placed in each case after the name of the institution :—

**Resident Medical Officers.**

Birmingham Borough Asylum. Clinical Assistant.—Board, no salary.
Brompton Consumption Hospital. Clinical Assistant.
Hull Royal Infirmary. Junior Assistant House Surgeon, fully qualified.—£50 with board.
North Eastern Hospital for Children. Junior House Surgeon. Doubly qualified.—£70.
Nottingham Medical Institution. Assistant. Doubly qualified.—£120.
Victoria Hospital for Children. Doubly qualified.—£40.
West London Hospital. House Surgeon. Registered.—Board, lodging, and attendance.
Wolverhampton General Hospital. Assistant. One qualification preferred.—Board, etc.

**Non-Resident Medical Officers.**

Redhill Reformatory.—£75.
Royal Maternity Charity. Physician.—£60.

**Matrons.**

Hospital for Incurables, Manchester.—£65.
Liverpool Northern Hospital.—£70, increasing to £100.

**Trained Nurses.**

Burton-on-Trent Nursing Institution. One. Certificated.
Hampstead Home Hospital. Two.—£16 to £20, with uniform.
Leeds District Nurses' Home. One.—£25 with board, etc.
Liverpool Northern Hospital. Head. £28 with uniform.
Merthyr Tydvil. Assistant-Surgical.
Mold Cottage Hospital. One.
Monsall Fever Hospital, Manchester. Several.—£25, all found.
Nottingham Nursing Institution. Several.
Suffolk General Hospital. Night. £20, board and uniform.
Tyrrell Cottage Hospital, Ilfracombe. Assistant Nurse (Lady). £24, with uniform.

**Probationers.**

Monsall Fever Hospital. Several.—£15 to £20, all found.
Grimsby Hospital. Two.—Entrance fee £5.

---

THE Committee of the French Hospital have fixed the 6th February next for their annual banquet, which will take place at Willis's Rooms, and will probably, as was the case last year, be presided over by His Excellency the French Ambassador, M. Waddington.

---

AT the fifteenth annual meeting of the subscribers to the Guest Hospital, Dudley, Mr. Poole (the secretary) presented the report, which stated that the expenditure had exceeded the income to the extent of £305 3*s.*, and that this addition to the already existing deficiency made up a total debt of £1,138 5*s.* 8*d*. The income from all sources for the past twelve months amounted to £3,023 14*s.*, and the committee had done all in their power to keep the expenditure within this amount, but without success.

---

IT is proposed to build a Cottage Hospital at Melton, to commemorate the jubilee year of Her Majesty's reign. The want of such an institution has been long felt, for only *bonâ fide* paupers are admitted to the Union Infirmary, and the nearest hospital is at Leicester, fully fifteen miles off.

---

THE annual meeting of the Driffield Cottage Hospital was held on the 17th ult. in the hall of the Mechanics' Institute, Mr. R. Holtby of Nafferton, being chairman. The expenditure for the year was stated to have been £212, and the receipts £226, so that there was a favourable balance of £14. During the past twelve months twenty-seven in- and thirty out-patients have received attention at the hospital.

---

THE Wrexham Philharmonic Society lately gave a very successful concert in the Public Hall, for the benefit of the Wrexham Infirmary, under the presidency of Mr. Archibald Peel. The choir of 150 voices was conducted by the Rev. C. Hylton Stewart, precentor of Chester Cathedral, and the proceedings were highly successful.

---

THE physicians of the Western Infirmary, Glasgow, invited Madame Marie Roze one day last week, to pay a visit of inspection to their hospital. The great vocalist readily complied, and spent a considerable time in the wards of the institution. Before leaving, one of the physicians suggested to Madame Roze that perhaps she would favour the inmates with a song. So all the patients, who were well enough to leave their wards, were taken into the large hall to hear the "Queen of Song." The patients were delighted with the treat, and one old lady cried out, in the exuberance of her gratitude, "You've done me more good than all the doctors."

---

AT a meeting of the Metropolitan Asylums Board on Saturday the 20th ult., a letter from the Local Government Board was read, nominating Professor Corfield and Dr. Seaton as members of the Board of Managers. The usual report was read, from which it appeared that 159 fever cases had been received during the preceding fortnight at the Eastern, Western, and South-Eastern Asylums. There remained under treatment 722 cases, or an increase of 45 on those reported a fortnight before. There were only two cases of small-pox in the hospital ships, and no fresh ones had been received.

---

AT the same meeting Captain Douglas Galton brought up the report of a committee on the question of building a permanent small-pox hospital at Darenth, on the Gore Farm estate, which had been bought for this purpose, with the consent of the Local Government Board. The committee recommended that brick huts should be erected on the approved site, to accommodate 560 patients, and they estimated the cost of these buildings at £84,000. There was some difference of opinion as to the adoption of this report by the Board, and eventually the consideration of the matter was adjourned.

---

A WEALTHY old gentleman, who is desirous of prolonging his days to the utmost possible extent has, saith rumour, recently entered into an agreement with his physician of a somewhat novel character. The patient will pay the physician each month, while he keeps him well, a sum equivalent to the fee for a daily visit, but as soon as he falls ill the doctor will attend him without a fee! The patient has just passed the grand climacteric, and believes he will eventually win the posthumous honours of centenarianism.

The Society of Medical Officers of Health has adopted a resolution, to the effect that it is desirable for local sanitary authorities to disinfect, free of charge, public cabs in which patients suffering from infectious diseases have been conveyed to the hospitals, and that a certificate of disinfection should be given to the driver. The attention of Sir Charles Warren, Commissioner of Metropolitan Police, has been drawn to the subject.

The late M. Kolomnin seems to have forgotten that it is not in mortals to command success. This distinguished Russian surgeon, who was a professor at St. Petersburg Surgical Academy, has taken his own life through chagrin at having failed in a dangerous surgical operation.

The Brompton Consumption Hospital has recently received some welcome legacies, including £300 from Miss Sprot, £50 from Miss Cayley, £1,000 from Miss Nightingale, £50 from the Rev. W. H. Edmeades, £20 from Miss Keating, £100 from Mr. G. K. Smith, and £300 from Mr. C. G. du Pré. As usual, each winter the demands on this institution are much in excess of its resources. From the report of the Committee of Management, read by Mr. Dobbin at the Court of Governors held last week, it appears that there are now no less than 232 applicants waiting admission, and the list is still growing.

Consumption has not inaptly been termed the "plague of England." Our climate—especially in the winter months—fosters the disease to such an extent that it has been estimated there are no less than 80,000 persons in this country who are constantly suffering from the fell disease. The demands, as we have said, upon the Brompton Consumption Hospital, are becoming more and more urgent, but the funds are still sadly in arrear of the needs of this institution.

Owing to the increased number of patients at the Chichester Infirmary during the past as compared with the previous year (the daily average being 47.12 as against 32.5), and also to the marked diminution in the life subscriptions (£21 as against £131 10*s*.), the deficit in accounts has increased by about £150, and forms the subject of serious anxiety to the committee. A large amount of work in repairs and sanitary improvements has also been found necessary. Mr. Mark Judge having recommended that the sewage shall for the future be disposed of by a process of soakage through porous drain-pipes placed under the lawn and garden, this advice has been adopted by the governing body, and the works will be commenced forthwith.

Mr. F. Maitland Moir, in a letter to the local press, appeals to the charitably disposed, for gifts of cast-off clothing for the convalescent patients at the City Hospital for Children, Cunnigarhill, Aberdeen. During a recent visit to the wards, Mr. Moir was "much struck by the fearfully ragged and dilapidated state of the clothing of some of the convalescents, which was actually in tatters," and he points out that those recovering from serious illness are, from their enfeebled condition, very liable to catch cold. Cannot some of our Scotch readers help in this matter?

One of the new wards of the Salford and Pendleton Hospital, the trustees of which held their fifty-ninth annual meeting on the 17th ult., is now occupied, and it is hoped that the whole of the new buildings will be completed by March next. The sum of £11,000 has been paid to the contractors on account of work done up to the present time.

The past year has been one of great anxiety to the authorities of the East London Hospital for Children at Shadwell. The amount of disease prevalent among children in the Shadwell district has been unusually large, and the extreme poverty and destitution of the neighbourhood render complete success in grappling with illness doubtful, if not impossible, since the misery and wretchedness of the children's homes cannot fail to greatly neutralise such benefit as they have received from their treatment in the hospital. The wealthy might generously send substantial monetary gifts to this valuable hospital.

The annals of our hospitals afford numerous instances of strange utterances by patients, and that, too, in cases where there is no reason to suspect a disordered mental condition. "Do you feel cold to-day?" repeatedly asked a female patient of the nurses at one of the large hospitals on one of the hottest days in the month of August last. A very remarkable circumstance is recorded many years ago of a boy, named John Scott Mayne, who lay sick with the cholera in the wards of the Royal Free. He seemed at the point of dissolution, when the doctors ordered a saline injection. Before the operation was over, the clock in the hospital struck the hour of eleven. To the bewilderment of everyone present, the boy asked, "What o'clock is it?" One of the nurses replied "ten." "No, it is not," responded the boy, with a hurried enunciation of the words, "it's eleven, for I counted it." The boy recovered, and in the course of a few days left the Royal Free Hospital fully restored to health.

Despite the long-continued depression in trade, it is satisfactory to note that some, at least, of our charities are managing to carry on their good work with undiminished resources. The Sunderland and Bishopwearmouth Infirmary, for example, has been able to issue a very favourable report for the year ending 30th June last. The ordinary income for the twelve months was £5,643; the year before it was £5,409, so that there was an actual increase of £234, while the extraordinary income was swelled by a bequest of £590. The Sunderland Infirmary is dependent upon voluntary contributions for very nearly the whole sum required for its annual expenditure.

The learned centenarian, Professor Chevreul, according to the *Sanitary Record*, offers a striking example of the connection between longevity and moderate living. A Paris contemporary states that every day M. Chevreul's breakfast consists of two eggs, a slice of chicken pasty made by his own cook, and a pint of *café au lait*. His dinner is also unvaried, and daily consists of tapioca soup with grated cheese, a cutlet, a bunch of grapes, cheese, and three glasses of water. M. Chevreul never eats fish nor drinks wine.

